# A cell type–specific approach to elucidate the role of miR-96 in inner ear hair cells

**DOI:** 10.3389/fauot.2024.1400576

**Published:** 2024-05-09

**Authors:** Kathleen Gwilliam, Michal Sperber, Katherine Perry, Kevin P. Rose, Laura Ginsberg, Nikhil Paladugu, Yang Song, Beatrice Milon, Ran Elkon, Ronna Hertzano

**Affiliations:** 1 Section on Omics and Translational Science of Hearing, Neurotology Branch, National Institute on Deafness and Other Communication Disorders, National Institutes of Health, Bethesda, MD, United States,; 2 Department of Human Molecular Genetics and Biochemistry, Tel Aviv University School of Medicine, Tel Aviv University, Tel Aviv, Israel,; 3 Department of Otorhinolaryngology Head and Neck Surgery, University of Maryland School of Medicine, Baltimore, MD, United States,; 4 Institute for Genome Sciences, University of Maryland School of Medicine, Baltimore, MD, United States

**Keywords:** miR-96, diminuendo, *Mir96* ^*Dmdo*^, hair cell, RNA-seq, inner ear, supporting cell, miRNA

## Abstract

**Introduction:**

Mutations in microRNA-96 (miR-96), a microRNA expressed within the hair cells (HCs) of the inner ear, result in progressive hearing loss in both mouse models and humans. In this study, we present the first HC-specific RNA-sequencing (RNA-seq) dataset from newborn *Mir96*^*Dmdo*^ heterozygous, homozygous mutant, and wildtype mice.

**Methods:**

Bulk RNA-seq was performed on HCs of newborn *Mir96*^*Dmdo*^ heterozygous, homozygous mutant, and wildtype mice. Differentially expressed gene analysis was conducted on *Mir96*^*Dmdo*^ homozygous mutant HCs compared to wildtype littermate controls, followed by GO term and protein-protein interaction analysis on these differentially expressed genes.

**Results:**

We identify 215 upregulated and 428 downregulated genes in the HCs of the *Mir96*^*Dmdo*^ homozygous mutant mice compared to their wildtype littermate controls. Many of the significantly downregulated genes in *Mir96*^*Dmdo*^ homozygous mutant HCs have established roles in HC development and/or known roles in deafness including *Myo15a, Myo7a, Ush1c, Gfi1*, and *Ptprq* and have enrichment in gene ontology (GO) terms with biological functions such as sensory perception of sound. Interestingly, upregulated genes in *Mir96*^*Dmdo*^ homozygous mutants, including possible miR-96 direct targets, show higher wildtype expression in supporting cells compared to HCs.

**Conclusion:**

Our data further support a role for miR-96 in HC development, possibly as a repressor of supporting cell transcriptional programs in HCs. The HC-specific *Mir96*^*Dmdo*^ RNA-seq data set generated from this manuscript are now publicly available in a dedicated profile in the gene expression analysis resource (gEAR-https://umgear.org/p?l=miR96).

## Background and summary

Sensorineural hearing loss (SNHL), doubles in incidence every decade of life, affecting 50% of people aged 70 and older (Lin et al., 2011). The final common pathway of most forms of SNHL involves the loss of the cochlear sensory cells, named hair cells (HCs). HCs are postmitotic specialized epithelial cells responsible for detecting and converting sound waves into electrical signals that are transmitted to the central nervous system (Fettiplace, 2017). Sound detection is mediated by stereocilia at the apical surface of the HCs (McGrath et al., 2017). It is estimated that mutations in more than 1,000 genes underlie sensorineural hearing loss (Ingham et al., 2019).

Point mutations in *MIR96/Mir96*, which encodes miRNA 96 (miR-96), are associated with progressive hearing loss in both humans and mice (Lewis et al., 2009; Ngeles Mencía et al., 2009; Kuhn et al., 2011; Soldà et al., 2012; Chen et al., 2014). The diminuendo mutant mouse (*Mir96*^*Dmdo*^) contains a point mutation in the seed region of miR-96 that results in the transversion of an adenosine (A) to a thymine (T) (Lewis et al., 2009). *Mir96*^*Dmdo*^ homozygous mutant mice (*Mir96*^*Dmdo/Dmdo*^) are profoundly deaf at birth and exhibit abnormal inner and outer HC (IHC and OHC, respectively) stereociliary bundles, smaller postnatal HC size, abnormal HC ion currents, immature ribbon synapses, and abnormalities of the auditory hindbrain anatomy and physiology (Lewis et al., 2009; Kuhn et al., 2011; Chen et al., 2014; Schlüter et al., 2018). *Mir96*^*Dmdo*^ heterozygotes exhibit an intermediate phenotype characterized by progressive hearing loss and progressive disorganization of their stereociliary bundles (Lewis et al., 2009). Similarly, specific deletion or misexpression of some or the entire miR-183/96/82 cluster in mice results in disarrayed stereociliary bundles in both IHCs and OHCs, as well as profound hearing loss (Fan et al., 2017; Geng et al., 2018; Weston et al., 2018; Lewis et al., 2021).

Previous studies have explored the mechanistic pathway and potential targets of miR-96 in the inner ear. Microarray studies of the organ of Corti identified the downregulation of genes associated with HC maturation in the *Mir96*^*Dmdo/Dmdo*^mice (Lewis et al., 2009, 2016). As miRNAs are known to directly inhibit their target genes, the downregulation of genes associated with HC maturation may be either a secondary effect from the loss of function of miR-96 target genes, reflecting a maturation arrest, or a direct effect from a change in the binding specificity of the novel *Mir96*^*Dmdo*^.

Although sequencing intact tissues, such as the organ of Corti, facilitates obtaining organ-specific transcripts for sequencing, it hinders the ability to detect cell-specific changes in gene expression due to signal averaging (Hertzano et al., 2021). As a result, particularly for cell types with lower abundance, it may be difficult to detect changes in the expression of genes that are also expressed in other cell types in the same tissue. For example, detecting changes in the expression of *Sox2* in the developing HCs may not be feasible via bulk RNA sequencing (RNA-seq) of the entire sensory epithelia, as during development it is expressed in multiple inner ear cell types (Hume et al., 2007). This limitation extends to other supporting cell (SC)–expressed genes that, earlier in development, are also expressed in HCs (e.g., Hayashi et al., 2010).

**FIGURE 1:**
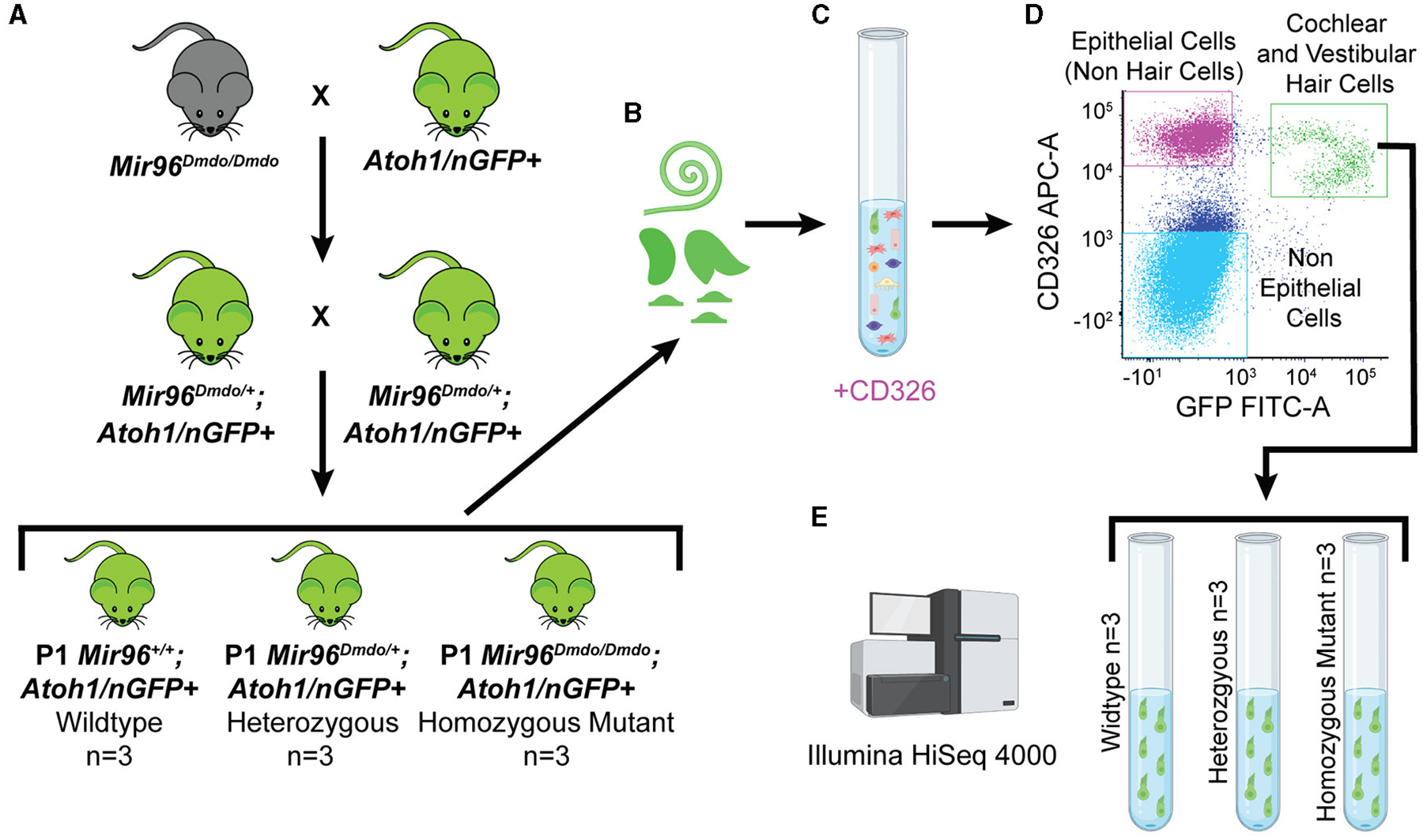
*Mir96*^*Dmdo*^ hair cell–specific bulk RNA sequencing. **(A)** Cross between *Mir96*^*Dmdo*^ mice and the *Atoh1/nGFP*+ mice to generate *Mir96*^*Dmdo*^; *Atoh1/nGFP*+ mice. **(B)** Cochleae and vestibular organs of *Mir96*^*Dmdo*^;*Atoh1/nGFP*+ wild-type, heterozygous, and homozygous mutant mice harvested at P1 and dissociated. **(C, D)** Staining of cochleae and vestibular organs of P1 *Mir96*^*Dmdo*^;*Atoh1/nGFP*+ mice with anti-CD326, an epithelial marker, allows for isolation of cochlear and vestibular hair cells (HCs; CD326- and GFP-positive gated together as a population, seen in green), epithelial non-HCs (CD326-positive gated as a population, seen in magenta), and non-epithelial cells (CD326- and GFP-negative gated together as a population, seen in light blue) using fluorescence-activated cell sorting. **(E)** Bulk RNA sequencing performed on P1 wild-type (*n* = 3), heterozygous (*n* = 3), and homozygous mutant (*n* = 3) *Mir96*^*Dmdo*^;*Atoh1/nGFP*+ HC samples. Created with Biorender.com.

To specifically isolate HCs from *Mir96*^*Dmdo*^ mice, *Mir96*^*Dmdo*^ HCs were fluorescently labeled by crossing *Mir96*^*Dmdo*^ mice to *Atoh1*/nGFP+ mice (*Mir96*^*Dmdo*^*;Atoh1/*nGFP+). Auditory and vestibular HCs were sorted from P1 *Mir96*^*Dmdo*^*;Atoh1*/nGFP+ wild-type, heterozygous, and homozygous mutant mice and underwent expression analysis by bulk RNA-seq (Figure 1). This data set identified 215 upregulated genes and 428 downregulated genes (fold-change > 1.5; false discovery rate [FDR] < 10%) in *Mir96*^*Dmdo*^ homozygous mutants compared to wild-type littermates. Genes essential in HC development and known deafness genes were identified as significantly downregulated in *Mir96*^*Dmdo*^ homozygous mutants while not previously identified as such in P4 and P0 *Mir96*^*Dmdo*^ whole organ of Corti microarray data sets (Lewis et al., 2009, 2016). Interestingly, several of the significantly upregulated genes identified in this HC-specific *Mir96*^*Dmdo*^ RNA-seq data set are enriched in SCs, some of which contain a conserved miR-96 seed in their 3′ untranslated region (UTR) region and are predicted miR-96 targets. These results suggest that miR-96 may function to repress the expression of SC-expressed genes in developing HCs.

This *Mir96*^*Dmdo*^ HC-specific RNA-seq data set is currently the only data set available for evaluating the regulatory cascade of *Mir96*^*Dmdo*^ specifically in HCs. It provides the opportunity to investigate and identify target genes of miR-96 that may otherwise have been undetected in data sets originating from inner ear tissues rather than an HC-specific analysis. This data set can be analyzed alongside other available data sets from the ear field and/or data sets focused on miR-96 for an opportunity to cross-reference gene expression data related to the regulatory pathways of miR-96 in inner ear development. These data are fully available for download, browsing, and analysis in the Gene Expression Analysis Resource (gEAR: umgear.org) via the following link: https://umgear.org/p?l=miR96 (Orvis et al., 2021). Identifying novel target genes of miR-96 is necessary to better understand genetic pathways involved in hearing loss caused by mutations in *Mir96/MIR96*. This will assist in determining the best mechanism to target for future precision medicine therapeutic intervention and molecularly assess the efficacy of therapeutic interventions.

## Materials and methods

### Animals

The diminuendo *Mir96*^*Dmdo*^ (C3HeB/FeJ) mice, with an A>T substitution within the seed region of *Mir96*, were generously provided by Dr. Karen Steel from the Wellcome Trust Sanger Institute, Hinxton, UK (Lewis et al., 2009). *Mir96* ^*Dmdo/Dmdo*^ mice were crossed with Atoh1/nGFP+ (C57BL6) mice, also known as *Math1/nGFP* mice, previously provided to us by Dr. Jane E. Johnson from the University of California, San Francisco. This cross generated *Mir96*^*Dmdo/+*^; *Atoh1/nGFP+* breeders, with a heterozygous A>T substitution in the seed region of miR-96 and GFP expression matching the spatiotemporal expression patterns of *Atoh1* (Lumpkin et al., 2003). All procedures were performed according to the National Institutes of Health Guide for the Care and Use of Laboratory Animals, with the approval of the Institutional Animal Care and Use Committee at the University of Maryland, Baltimore (protocol numbers 1209008, 1112005, 1015003, and 0918005).

### Genotyping

The genotyping of *Mir96*^*Dmdo*^*;Atoh1/nGFP* mice was performed on tail tips or ear notches through TaqMan real-time polymerase chain reaction (qPCR) assays. The following primers and reporters were used to genotype *Mir96*: forward primer (5′ CCTGTTCCAGTACCATCTGCTT 3′), reverse primer (5′ AGCGGAGAGACACAAGCAAA 3′), reporter 1 (5′ AATGTGCTAGTGCCAAAA 3′), and reporter 2 (5′ ATGTGCTAGAGCCAAAA 3′) and to genotype Atoh1/nGFP: forward primer (5′ CGTCGTCCTTGAAGAAGATGGT 3′), reverse primer (5′ CACATGAAGCAGCACGACTT 3′), and reporter 1 (5′ CATGCCCGAAGGCTAC 3′) (Transnetyx^©^).

### Hair cell isolation

Inner ears from postnatal day (P) 1 wild-type (*n* = 3 mice), heterozygous (*n* = 3 mice), and homozygous mutant (*n* = 3 mice) *Mir96*^*Dmdo*^*;Atoh1/nGFP* mice were harvested in ice-cold 1× phosphate buffered saline (PBS). Cochleae and vestibular organs from both ears of each mouse were then harvested in ice-cold 1× PBS and placed into a well of a 48-well plate filled with 300 μL of 0.5 mg/mL thermolysin (one well per mouse), as previously described (Hertzano et al., 2011). Samples were then placed in a 37°C, 5% CO_2_ humidified tissue culture incubator for 20 min. The thermolysin (Sigma-Aldrich) was carefully removed, avoiding contact with any cochlear or vestibular tissue, and 250 μL of Accutase-enzyme cell detachment medium (Sigma-Aldrich) was pipetted into each well. All samples were placed in a 37°C, 5% CO_2_ humidified tissue culture incubator for 3 min. Tissue samples were then dissociated by trituration (15–20 repeats) using a 26-gauge blunt-end needle. This process was repeated two to three times until the cells appeared to be fully dissociated by direct visualization using an inverted tissue culture microscope. To deactivate the Accutase, 250 μL of IMDM + 10% fetal bovine serum was added to each well. Next, the cells were strained through 35 μm cell-strainer filter caps into 5 mL polystyrene round-bottom tubes (Falcon), spun down at 500 g at 4°C for 1 min, and kept on ice. To differentiate between GFP expressed in HCs and neurons, samples were stained with an antibody for the epithelial marker CD326 (anti-CD326 (Ep-CAM)-APC Conjugated, Biolegend, 1:2000). Cells positive for both CD326 and GFP represent HCs, whereas cells positive only for GFP represent neuronal cells (Elkon et al., 2015).

Cells were sorted with a BD FACSAria^™^ II flow cytometer at the University of Maryland School of Medicine Flow Cytometry Core to collect double-positive cells (GFP-positive, CD326-positive) that are cochlear and vestibular HCs. The total numbers of cochlear and vestibular HCs obtained were 3,900–5,000 for *Mir96*^*Dmdo*^ wild-type mice replicates, 3,700–9,500 for *Mir96*^*Dmdo*^ heterozygous mice replicates, and 1,300–4,600 for *Mir96*^*Dmdo*^ homozygous mutant samples.

### RNA extraction and sequencing

RNA was extracted from sorted cochlear and vestibular HCs using the Direct-zol RNA MiniPrep Kit (Zymo Research). First, TRIzol^™^ LS (Invitrogen^™^) was added to each sample of HCs to 70% of the total volume. All following steps were conducted as outlined in the Direct-zol RNA MiniPrep Kit protocol, eluting RNA in 25 μL of nuclease-free water in the final step.

RNA integrity was measured at the Genomics Core Facility at the University of Maryland School of Medicine with a eukaryotic total RNA pico chip assay on the Agilent 2100 Bioanalyzer. Seven samples had an RNA integrity number (RIN) score >8, one wild-type replicate had a RIN of 6.3, and one *Mir96*^*Dmdo*^ homozygous mutant sample had a RIN of 7.8. All samples were included due to limited sample availability.

RNA-seq was conducted on all wild-type (*n* = 3 mice), heterozygous *n* = 3 mice), and homozygous mutant (*n* = 3 mice) *Mir96*^*Dmdo*^*;Atoh1/nGFP* HC samples (double-positive sorted cells) at the Institute for Genome Sciences Genomics Resource Center of the University of Maryland School of Medicine. Libraries were prepared from 2.75 to 5.43 ng of RNA from each sample using the NEBNext Ultra Directional RNA Kit per the manufacturer’s instructions with a total of 22 polymerase chain reaction (PCR) cycles, except for a 5-min fragmentation time instead of 15 min and a solid-phase reversible immobilization (SPRI) bead size selection before PCR. Libraries were sequenced on an Illumina HiSeq 4000 sequencer, 0.11 lanes/sample, with 75 base pair (bp) paired-end reads. An approximate range of 56M−102M total reads were obtained for each sample.

### RNAseq normalization and expression analysis

The quality of the FASTQ files was evaluated with FastQC. The reads were aligned to the mouse genome (Mus_musculus.GRCm38) using HiSat (version HISAT2–2.0.4), and the number of reads that aligned to the coding regions was determined using HTSeq (Anders et al., 2015; Kim et al., 2015).

Our analysis was conducted on the 15,294 genes that were robustly detected in the data set, defined as those that were covered by a minimum of 20 reads in at least 2 of the 3 replicates of any genotype group (wild-type, heterozygous, and homozygous mutant *Mir96*^*Dmdo*^*;Atoh1/nGFP*+ HCs). The genes were annotated using BioMart annotations version 86 (Smedley et al., 2015). DEseq2 was used for all differential expression analysis, calling differentially expressed (DE) genes between genotype groups based on FDR-adjusted *p*-values (FDR < 10%) and a fold-change >1.5 (Love et al., 2014). Overall, in the comparison between *Mir96*^*Dmdo*^ homozygous mutant and wild-type HCs, 428 downregulated and 215 upregulated genes were detected. The volcano plot was generated by EnhancedVolcano version 1.18 (Blighe et al., 2018). Gene ontology (GO)–enrichment tests were performed using the clusterProfiler package, using the parameters: Ontology—biological process, *p*-value adjustment method – FDR, adjusted *p*-value cutoff – 5% (Wu et al., 2021). The network analysis was conducted using DOMINO (http://domino.cs.tau.ac.il/) after mapping mouse genes to their human orthologs, only including genes with one-to-one mapping (Levi et al., 2021, 2022).

### Hair cell and supporting cell expression analysis of upregulated genes in *Mir96*^*Dmdo*^ homozygous mutant hair cells

A P1 wild-type mouse cochlea scRNA-seq data set (Kolla et al., 2020) was downloaded via umgEAR.org (including metadata for cell cluster identification) and analyzed via Seurat v4. Gene expression was pseudo-bulked with Seurat’s AverageExpression function (*Z*-score), and the genes significantly upregulated in *Mir96*^*Dmdo*^ homozygous mutant HCs were displayed via heatmaps (4 out of the 215 significantly upregulated genes were not included in the heatmap visualization or analysis of the wild-type expression of these genes in HCs and SCs as they were not found in the Kolla et al., 2020 data set). A differential expression analysis between wild-type P1 HCs and SCs was performed with DESeq2 via Seurat’s FindMarkers function. Box plots displaying Log_2_ fold change (unpaired) and pseudo-bulked average expression (paired) between HCs and SCs were created in R with packages ggplot2 and ggpubr. The Wilcoxon rank-sum test (unpaired box plot) and Wilcoxon signed-rank test (paired box plot) were used for significance testing in R.

## Results

**FIGURE 2:**
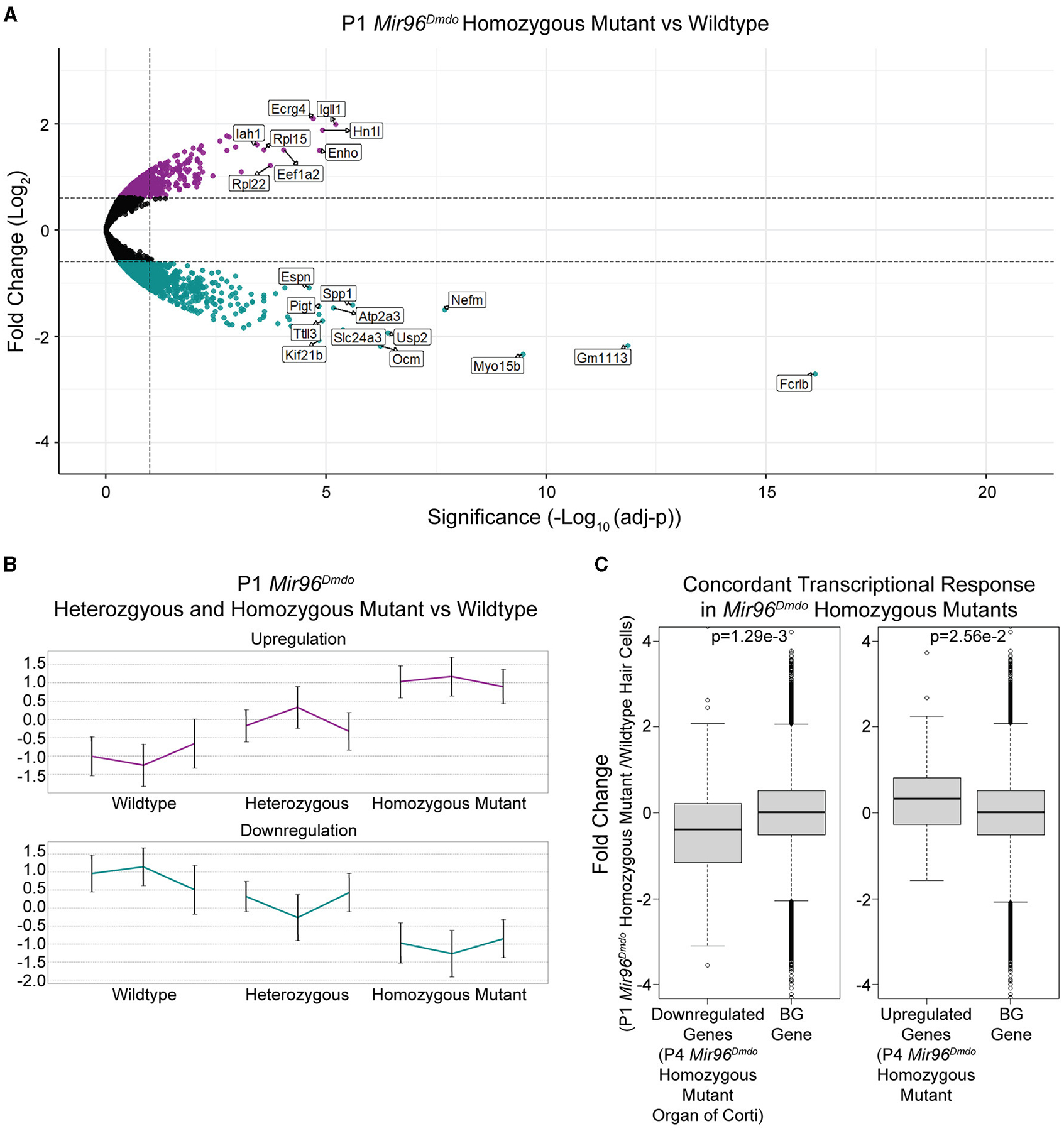
*Mir96*^*Dmdo*^ hair cell differentially expressed gene analysis. **(A)** Volcano plot for the differential expression analysis between *Mir96*^*Dmdo*^ homozygous mutants and wild-type controls. Significantly downregulated and upregulated genes (false discovery rate < 10%, fold-change > 1.5) are colored in cyan and purple, respectively. Selected top-scoring genes are highlighted. **(B)** Standardized expression levels of the differentially expressed genes as measured in each sample of the three genotypes: *Mir96*^*Dmdo*^ wild type, heterozygous, and homozygous mutants. Each cluster is represented by its mean pattern. Error bars indicate ± standard deviation. **(C)** The top 55 most strongly downregulated and top 37 most strongly upregulated genes in the P4 *Mir96*^*Dmdo*^ homozygous mutant organ of Corti microarray data set with a Log_2_fold-change cuto of 0.5 (Lewis et al., 2009) showed a corresponding significant change in expression in the P1 *Mir96*^*Dmdo*^ homozygous mutant hair cells (HCs) compared to controls in our data set. Each panel shows the comparison between the P4 *Mir96*^*Dmdo*^ homozygous mutant organ of Corti differentially expressed genes (Lewis et al., 2009) and a background set of genes consisting of all the other genes in our data set (Left: top 55 downregulated genes in Lewis et al., 2009, data set; Right: top 37 genes upregulated in Lewis et al., 2009, data set). The *Y*-axis shows the fold-change in expression measured in our P1 HC-specific *Mir96*^*Dmdo*^ RNA-seq data set (in Log_2_ scale). *p*-values were calculated using the Wilcoxon test: *p* = 1.29e-3 (downregulated genes in *Mir96*^*Dmdo*^ homozygous mutants) and *p* = 2.56e-2 (upregulated genes in *Mir96*^*Dmdo*^ homozygous mutants).

To identify genes mis-regulated in the *Mir96*^*Dmdo*^ HCs, RNA-seq was performed on HC samples from *Mir96*^*Dmdo*^wild-type, heterozygous, and homozygous mutant mice. *Mir96*^*Dmdo*^ heterozygous HCs were included in the transcriptomic analysis because *Mir96*^*Dmdo*^ heterozygous mice manifest an intermediate auditory phenotype. Overall, our transcriptomic data set contained 15,294 robustly detected genes, each gene with a minimum of 20 reads in at least 2 of the 3 biological replicates of any one of the genotypes: *Mir96*^*Dmdo*^wild-type, heterozygous, or homozygous mutant cochlear and vestibular HCs. Of the 15,294 genes, 13,022 were protein-coding genes, and 861 were annotated as lincRNAs (Supplementary Table 1). We conducted a differential expression analysis to identify genes whose expression levels were significantly altered in the *Mir96*^*Dmdo*^ homozygous mutant HCs compared to wild-type controls. This analysis detected 428 significantly downregulated and 215 significantly upregulated genes in the *Mir96*^*Dmdo*^ homozygous mutant HCs compared to wild-type control HCs (FDR < 10%, fold-change > 1.5; Figure 2A).

Additionally, we evaluated if the intermediate auditory phenotype of the *Mir96*^*Dmdo*^ heterozygous mice is also reflected in the gene expression profile of *Mir96*^*Dmdo*^ heterozygous HCs. Markedly, genes that were differentially expressed between the *Mir96*^*Dmdo*^ homozygous mutant and control HCs were partially up- or downregulated in the *Mir96*^*Dmd*o^ heterozygous HCs, corresponding to a midway change in expression (Figure 2B).

To examine concordance between the transcriptomic profiles of our cell-specific RNA-seq data set of P1 *Mir96*^*Dmdo*^HCs and the previously published microarray data set of P4 *Mir96*^*Dmdo*^ organ of Corti (Lewis et al., 2009), we focused on the most strongly upregulated (37) and downregulated (55) genes in the P4 *Mir96*^*Dmdo*^ homozygous mutant organ of Corti (Log_2_fold-change cutoff = 0.5; Lewis et al., 2009) and evaluated if the expression of these genes was concordantly up- and downregulated in our data set. We found that the differential expression of these 55 downregulated and 37 upregulated genes in *Mir96*^*Dmdo*^homozygous mutants compared to wild-type controls showed a highly consistent response between the two data sets (Figure 2C; downregulated genes: *p* = 1.29e-3; upregulated genes: *p* = 2.56e-2). Even with this overall similarity of the top responsive genes, we identified genes that were significantly up- or downregulated in our P1 HC-specific *Mir96*^*Dmdo*^ RNA-seq data set that were not significantly responsive in either the P4 or P0 *Mir96*^*Dmdo*^ organ of Corti microarray data sets (FDR < 5%; Lewis et al., 2009, 2016).

Testing the DE genes detected in our data set for enrichment for the GO categories, we determined biological processes that are compromised in the *Mir96*^*Dmdo*^ homozygous mutant HCs. The downregulated genes of *Mir96*^*Dmdo*^ homozygous mutant HCs were strongly enriched for genes that function in sensory perception of sound (FDR: 2.62E-05), synaptic transmission (FDR: 1.94E-03), and HC differentiation (FDR: 5.66E-03; Table 1), providing a molecular underpinning for the auditory phenotype observed in the *Mir96*^*Dmdo*^ homozygous mutant mice. However, in our data set, upregulated genes of *Mir96*^*Dmdo*^ homozygous mutant HCs function in many diverse biological processes, with no enrichment for any process.

**FIGURE 3:**
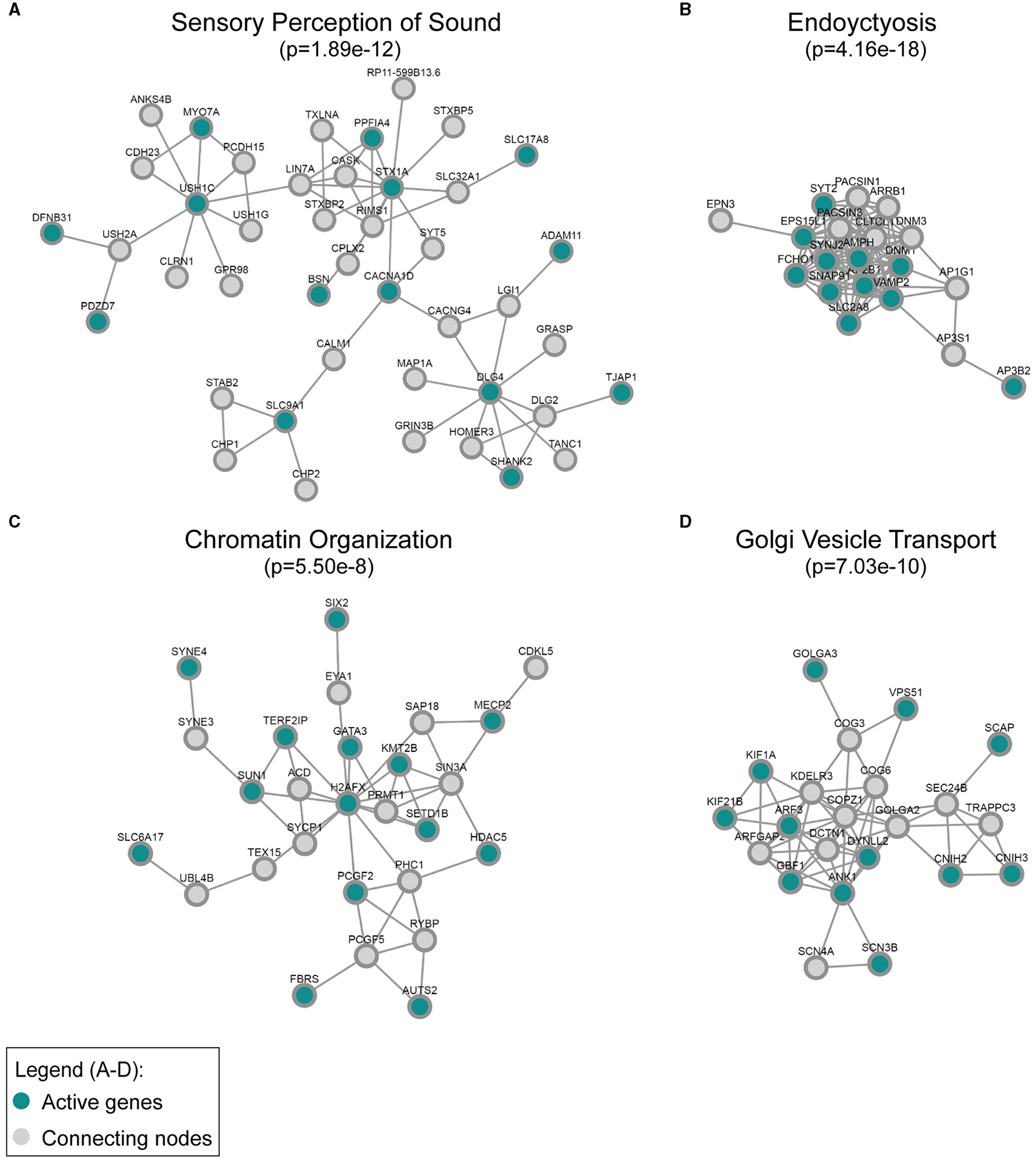
Active modules enriched for genes downregulated in the *Mir96*^*Dmdo*^ homozygous mutants were detected in the global human protein–protein interaction network. The most strongly enriched gene ontology category in each module, **(A)** sensory perception of sound, **(B)** endocytosis, **(C)** chromatin organization, and **(D)** Golgi vesicle transport, is indicated together with its false discovery rate–adjusted *p*-value. “Active” genes (in this case, genes downregulated in the *Mir96*^*Dmdo*^ homozygous mutants) are colored in cyan, while all genes colored in gray are connecting nodes (all of which are background genes of the P1 *Mir96*^*Dmdo*^ homozygous mutants and wild-type samples).

Additionally, we applied network analysis to the set of DE genes. We used our recently developed DOMINO algorithm. The input of the analysis consists of a large protein–protein interaction (PPI) network and the set of DE genes and DOMINO searches the PPI of sub-networks of interacting proteins that are enriched for the DE genes (Levi et al., 2021, 2022). Each such subnetwork (termed an “active module”) represents a functional unit of interacting proteins. Using the STRING (human) PPI (Szklarczyk et al., 2021), DOMINO identified four main active modules among the genes that were downregulated in the *Mir96*^*Dmdo*^ homozygous mutant HCs. In addition to the key module enriched for proteins that function in “perception of sound”, functional modules for “chromatin organization”, “endocytosis”, and “Golgi vesicle transport” were also detected (Figure 3).

miRNAs mainly exert a destabilizing effect on their target genes. miRNA target gene repression is initiated by the binding of the miRNA to *cis*-regulatory target sites that, for the majority, are embedded in the 3′ UTRs of target genes. Therefore, direct targets of miR-96 are expected to be repressed in wild-type tissue and, in turn, would have upregulated expression in the HCs of *Mir96*^*Dmdo*^ homozygous mutant compared to wild-type littermates. Consequently, seeking direct target genes of miR-96 in the mouse inner ear, we searched the genes upregulated in the *Mir96*^*Dmdo*^ homozygous mutant HCs for predicted miR-96 target sites that are conserved between human and mouse. Using TargetScan predictions, we found that 17% of the upregulated genes in the HCs of *Mir96*^*Dmdo*^ homozygous mutants (13 of the 77 genes in the upregulated set that were included in TargetScan DB) were predicted as miR-96 direct targets (Supplementary Table 1B; Agarwal et al., 2015). Notably, the proportion of predicted miR-96 direct targets among the upregulated genes was significantly higher than its proportion among the downregulated genes of *Mir96*^*Dmdo*^ homozygous mutant HCs (6%; 16 of the 240 genes in this set that were included in TargetScan DB, *p*-value for the difference = 0.007, chi-square test).

**FIGURE 4:**
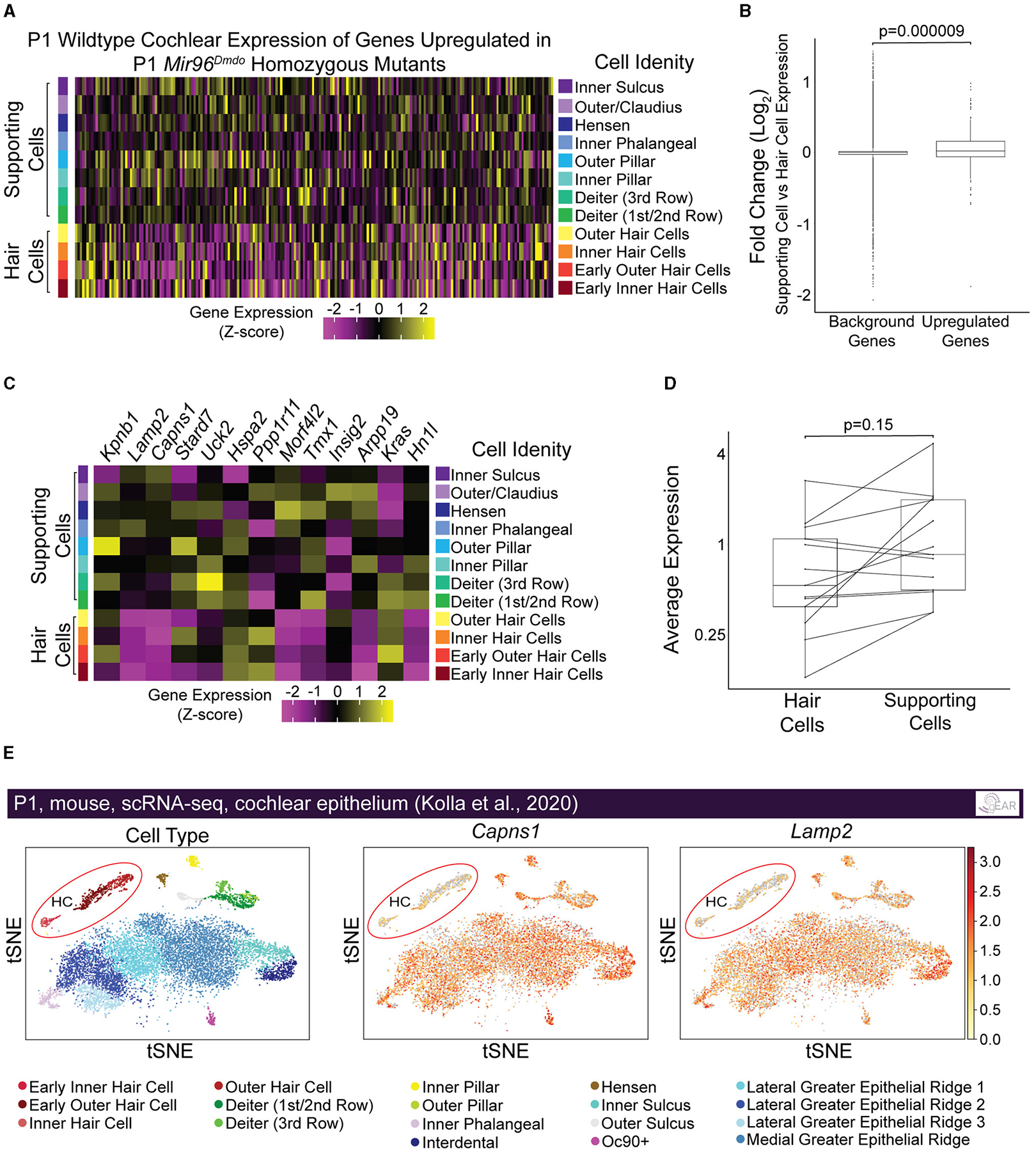
Genes upregulated in *Mir96*^*Dmdo*^ homozygous mutant hair cells are enriched in supporting cells compared with hair cells in inner ears of P1 wild-type mice. **(A)** Heatmap of pseudo-bulked gene expression (*Z*-scores) for all genes significantly upregulated in *Mir96*^*Dmdo*^ homozygous mutant hair cells (HCs) expressed in a wild-type P1 scRNA-seq data set. **(B)** Box plot displaying Log_2_ fold-change (supporting cells [SCs] vs. HCs; P1 wild-type mice) of all genes significantly upregulated in *Mir96*^*Dmdo*^ homozygous mutant HCs compared to background (all genes expressed in the data set). Wilcoxon rank-sum test, *p* = 0.000009. **(C)** Heatmap of pseudo-bulked gene expression (*Z*-scores) for genes significantly upregulated in *Mir96*^*Dmdo*^ homozygous mutant HCs predicted as direct targets of miR-96 expressed in a wild-type P1 scRNA-seq data set. **(D)** Paired box plot displaying pseudo-bulked average expression in HCs and SCs of the 13 genes significantly upregulated in *Mir96*^*Dmdo*^ homozygous mutant HCs predicted as direct targets of miR-96. Wilcoxon signed-rank test, *p* = 0.15. **(E)** Expression of *Capns1* and *Lamp2* visualized in tSNE space (adapted from umgear.org). All expression data shown in this figure are from a previously published P1 wild-type mouse cochlear epithelial data set (Kolla et al., 2020).

We hypothesized that genes targeted by miR-96 in HCs are more specifically and highly expressed in non-HC cell types, including SCs, and are repressed in HCs by miR-96 during HC differentiation. Therefore, we evaluated the expression of upregulated genes in *Mir96*^*Dmdo*^ homozygous mutant HCs, in P1 wild-type HCs and SCs from a previously published data set (Kolla et al., 2020). Indeed, we found that the genes upregulated in the *Mir96*^*Dmdo*^ homozygous mutant HCs, as a group, had significantly higher expression in wild-type SCs as compared to HCs, suggesting a role for miR-96 in suppressing genes more highly expressed in SCs than in HCs (Figures 4A, B, *p* = 0.000009; Supplementary Figure 1, *p* = 0.00002). We next analyzed the subgroup of upregulated genes with an miR-96 seed region in their 3′ UTR (i.e., predicted target genes, *n* = 13). Of these 13 candidate miR-96 targets, 6 showed a higher expression in SCs compared to HCs (Figures 4C, D, *p* = 0.15), among them *Capns1* and *Lamp2* (Figures 4C, E), suggesting a direct repressive effect for miR-96 on these genes in HCs.

## Discussion

Here, we shared the first HC-specific RNA-seq data set and analysis of *Mir96*^*Dmdo*^ mice to further characterize the gene regulatory network downstream of miR-96 in HCs. Previous work investigating the regulatory role of miR-96 in the inner ear used microarray data to analyze the expression of RNA extracted from the entire organ of Corti. This approach may limit the ability to identify changes in the expression of genes with an inner ear expression not limited to HCs (e.g., HCs and SCs or mesenchyme). With an HC-specific RNA-seq approach, we were able to identify significantly downregulated genes that were enriched for the GO term of sensory perception of sound, synaptic transmission, and sensory organ morphogenesis, which consisted of genes necessary for HC development and hearing, as well as known deafness genes. These genes included *Fbxo2, Myo15a, Myo7a, Otof, Spp1, Syt2, Ttll3*, and *Ush1c*, which were not identified as DE genes in either the P4 or the P0 *Mir96*^*Dmdo*^ microarray data set, indicating the need for an HC-specific approach to identify the differential expression of such genes in HCs. A fewer number of genes, including *Gfi1, Ocm*, and *Ptprq*, were previously identified as significantly downregulated in the P4 and/or P0 *Mir96*^*Dmdo*^ microarray data sets that were also significantly downregulated in this P1 HC-specific RNA-seq data set (Lewis et al., 2009, 2016). In addition, despite a similarity in HC gene expression in P0 and P1 HCs, the previously published P0 *Mir96*^*Dmdo*^ organ of Corti microarray data set only identified 18 significantly DE genes (Lewis et al., 2016). This further shows the benefit of using an HC-specific RNA-seq approach to identify a much larger number of DE genes and, in turn, potential targets of miR-96 in HCs at an earlier HC developmental time to better understand the progressive phenotype of the *Mir96*^*Dmdo*^ mice.

Our analysis further highlights the complementary roles of the GO and PPI analyses. While both analyses identified sensory organ development and HC differentiation as functions enriched in the downregulated genes, the PPI analysis further discovered enrichment for endocytosis, chromatin organization, and Golgi vesicle transport. Some of the genes within the PPI network enriched for endocytosis and chromatin organization have known roles in HC function and/or hearing, such as *Dnm1, Syt2*, and *Syne4* (Horn et al., 2013; Johnson et al., 2013; Herrmann et al., 2014; Neef et al., 2014; Masterson et al., 2018). Other proteins that interact with *DNM1, SYT2*, and *SYNE4* are likely excellent candidates to investigate as having a role in hearing as well. Interestingly, a number of the significantly downregulated genes of the PPI networks, including those enriched for chromatin organization and Golgi vesicle transport, have no known role in hearing, including *Kif21b, Scn3b*, and *Slc6a17*, and are highly and specifically expressed in HCs at P1 (Kolla et al., 2020). These genes may be interesting, novel candidates involved in HC development that may allow us to better understand miR-96’s role in hearing as well as these genes’ roles in the inner ear during development.

Prior research identified genes necessary for non-sensory cell development in the inner ear, including *Zeb1* and *Snai2*, as direct targets of miR-96 (Lewis et al., 2021). Here, we further show that genes upregulated in the *Mir96*^*Dmdo*^ HCs, as a group, have a higher expression in wild-type SCs as compared with HCs. Therefore, we hypothesize that the role of miR-96 in HCs is to repress the expression of genes with a broad expression pattern in the organ of Corti during early development. We must not rule out the possibility that some up- and downregulated genes in this data set may not be direct or indirect targets of miR-96 but, instead, are novel targets of the *Mir96*^*Dmdo*^ allele. Previous work of P4 and P0 *Mir96*^*Dmdo*^ organ of Corti microarray data sets has shown that *Ptprq*, a gene downregulated in our data set, contains the novel seed region of the *Mir96*^*Dmdo*^ mutation. This finding suggested that *Ptprq* is likely a target of the miR-96^*Dmdo*^ rather than a wild-type miR-96 target (Lewis et al., 2021).

An important limitation of this study is the inclusion of both cochlear and vestibular HCs to generate the RNA for bulk sequencing. While both the cochlear and vestibular HCs of the *Mir96*^*Dmdo*^ mice exhibit a similar phenotype, namely, abnormal stereocilia and HC degeneration (Lewis et al., 2009), previous studies have shown transcriptional differences between cochlear and vestibular HCs (Scheffer et al., 2015). Therefore, we analyzed the wild-type expression of the downregulated genes in our P1 *Mir96*^*Dmdo*^ RNA-seq data set in auditory and vestibular HCs to ensure these genes are, in fact, expressed and thus affected by the miR-96 mutation in both systems. We found that 375 of the 416 downregulated genes in our data set are expressed in wild-type P1/P2 cochlear and vestibular HCs (McInturff et al., 2018; Kolla et al., 2020; Kalra et al., 2023; 12 downregulated genes were not detected in the P1/P2 wild-type cochlear and vestibular HCs). Future studies could use scRNA-seq to analyze the differential role for miR-96 in auditory and vestibular HCs.

In addition to HCs, members of the miR-183/96/82 cluster, together and individually, play a role in the development of other sensory cells, including retinal cells and neurons (Davari et al., 2017; Peng et al., 2018; Zolboot et al., 2021; Urbán et al., 2023). Specifically, the miR-183/96/82 cluster is necessary for photoceptor differentiation and maintenance in mice (Zhang et al., 2020; Xiang et al., 2022). This suggests that some targets of miR-96 and/or the miR-183/96/82 cluster may be shared between the auditory and visual systems. Importantly, when searching for similarities and differences in gene expression patterns between data sets of perturbation of the miR-183/96/82 cluster across systems, up- and downregulated genes must be analyzed separately. Upregulated genes would be candidates for direct targets of the miR-183/96/82 cluster or one of its members. By comparison, downregulated genes, as shown in our data set, can reflect a developmental arrest rather than a direct effect of the miR-96 mutation. For example, *Ush1c* was significantly downregulated in our P1 *Mir96*^*Dmdo*^ HC RNA-seq data set. Mutations in USH1C affect both the auditory and visual systems, causing profound bilateral deafness, vestibular dysfunction, and progressive retinitis pigmentosa. However, mutations in *MIR96*, to date, have not been associated with syndromic forms of hearing loss – supporting the hypothesis that either the downregulation of *Ush1c* with disruption of the miR-183/96/82 cluster signaling is cell type–specific or the downregulation of *Ush1c* in the mutant HCs reflects a maturation arrest. It is also possible that deeper phenotyping of patients with mutations in *MIR96* could reveal a retinal phenotype.

This study further highlights the importance of the non-coding genome in gene regulation of the inner ear. Many advancements in technology, such as improved RNA sequencing, miRNA sequencing, and enhanced bioinformatics tools, have allowed us to better identify mutations in the non-coding genome that lead to hearing loss as well as understand elements of the non-coding genome involved in inner ear development and auditory function (Schrauwen et al., 2016; Avraham et al., 2022). The non-coding genome consists of many elements, including enhancers, long non-coding RNA (lncRNA), and miRNAs. LncRNAs, such as *Lockd, Dlx1as, Emx2os*, and *Soxot*, are of interest because they are located in unresolved deafness-associated loci (Ushakov et al., 2017). Additionally, recent studies have begun to hypothesize roles for some lncRNAs in the inner ear, including *Gas5*, which may regulate the *Notch1* pathway (Koffler-Brill et al., 2021). miRNAs have specific spatiotemporal expression in the auditory and vestibular systems, with some having known roles in inner ear development and function (Friedman et al., 2009; Rudnicki et al., 2014; Mahmoudian-sani et al., 2017). This includes the miR-183/92/182 cluster discussed in this study, as well as miR-199a, miR-18a, miR-15a, and miR-431 (Friedman et al., 2009; Fan et al., 2016). We can benefit from comparing the regulatory cascades of different miRNAs and lncRNAs to better understand their role in the inner ear and possible compensatory ability for one another and identify targets for potential therapeutics in patients with mutations affecting the expression/function of these genes.

## Supplementary Material

Supplementary Figure 1

Supplementary Table 2

Supplementary Table 1

## Figures and Tables

**TABLE 1:** Downregulated genes of *Mir96*^*Dmdo*^ homozygous mutant hair cells were enriched for biological processes including sensory perception of sound, synaptic transmission, ion homeostasis, sensory organ morphogenesis, and hair cell differentiation.

GO term	FDR	Genes
Sensory perception of sound	2.62e-05	*Strc, Tmc2, Pdzd7, Loxhd1, Dnm1, Myo15, Espn, Srrm4, Atp2b2, Whrn, Tomt, Ush1c, Myh14, Tmprss3, Otof, Myo7a, Espnl, Ptprq, Cacna1d, Slc17a8*
Synaptic transmission	1.94e-03	*Car7, Kcnc3, Calb1, Stx1a, Grm4, Slc12a5, Cnih3, Grid2ip, Cux2, Shank2, Egr3, Rimbp2, Prkcz, Dnm1, Shisa6, Syt16, F2r, Atp2b2, Dlg4, Gabbr1, Syt2, Vamp2, Brsk1, Otof, Asic1, Htt, Mecp2, Btbd9, Snap91, Cnih2, Cplx2, Slc17a8, App*
Ion homeostasis	1.94e-03	*Ocm, Slc24a3, Car7, Hrh3, Calb1, Cln3, Atp2a3, Slc12a5, Car12, Ptgir, Slc25a27, Slc25a23, Atp13a2, Atp6v0a1, Efhc1, Slc4a3, Slc9a1, Ank1, F2r, Atp2b2, Mcu, Sfxn2, Dlg4, Slc22a17, Gstm7, Scn3b, Tmprss3, Wnk2, Gpr4, Htt, Btbd9, Prkaa2, Atp2b1, Mllt6, App*
Sensory organ morphogenesis	4.89e-03	*Strc, Calb1, lrx5, Bmp7, Pdzd7, Miat, Six2, Myo15, Gata3, Atp2b2, Whrn, Ahi1, Ush1c, Vangl2, Casz1, Mfn2, Myo7a, Ptprq, Tbx2, Gfi1*
Hair cell differentiation	5.66e-03	*Strc, Pdzd7, Atp2b2, Whrn, Tomt, Ush1c, Myo7a, Ptprq, Gfi1*

## Data Availability

The datasets presented in this study can be found in online repositories. Raw fastq files for all data have been deposited in the Gene Expression Omnibus (GEO) -NCBI (Gwilliam et al., 2024, GEO GSE255796). Information on each of the *Mir96*^*Dmdo*^ wild-type, heterozygous, and homozygous mutant HC biological replicates of these data is outlined in Supplementary Table 2. A list of all significantly up- and downregulated genes in the HCs of *Mir96*^*Dmdo*^ homozygous mutant mice, and whether they contain the miR-96 binding motif within the 3’UTR, can be found in Supplementary Table 1B. Additionally, these data are publicly available through gEAR (umgear.org) for visualization and further analysis through the following link (https://umgear.org/p?l=miR96). We used a previously published microarray dataset (Lewis, 2009, ArrayExpress E-TABM-489) to compare the differentially expressed genes of HCs of our dataset to those of the organ of Corti of the same diminuendo (*Mir96*^*Dmdo*^) mouse model. Additionally, we used a previously published single cell RNA-sequencing (scRNA-seq) dataset (Kelly et al., 2020, GEO GSE137299) to evaluate the P1 wildtype expression of genes significantly upregulated in P1 *Mir96*^*Dmdo*^ homozygous mutant HCs in SCs compared with HCs.
