# Supplementary material for: A cell type–specific approach to elucidate the role of miR-96 in inner ear hair cells
Source: Front Audiol Otol. Author manuscript; Available in PMC 2024 May 31. (PMC11141775; doi:10.3389/fauot.2024.1400576)
Supplement: Supplementary Figure 1 [file NIHMS1993125-supplement-Supplementary_Figure_1.pdf]

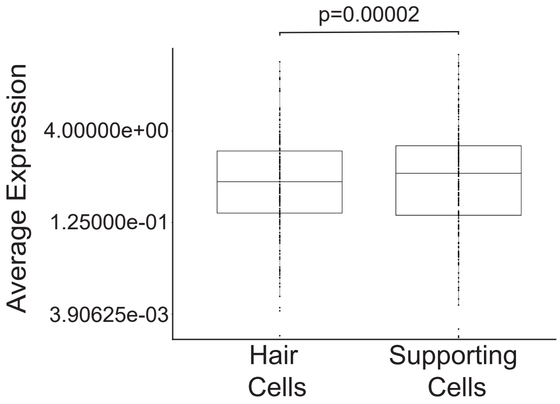

Supplementary Figure 1. Upregulated genes in *Mir96*^{Dmdo} homozygous mutant hair cells have higher expression in supporting cells of P1 wildtype cochlea compared with hair cells. Paired boxplot displaying pseudo-bulked average expression in HCs and SCs of all genes significantly upregulated in *Mir96*^{Dmdo} homozygous mutant HCs. Wilcoxon signed-rank test, p=0.00002.
